# Isoprinosine as a foot-and-mouth disease vaccine adjuvant elicits robust host defense against viral infection through immunomodulation

**DOI:** 10.3389/fcimb.2024.1331779

**Published:** 2024-03-06

**Authors:** Hyeong Won Kim, Mi-Kyeong Ko, Seokwon Shin, So Hui Park, Jong-Hyeon Park, Su-Mi Kim, Min Ja Lee

**Affiliations:** Center for Foot-and-Mouth Disease Vaccine Research, Animal and Plant Quarantine Agency, Gimcheon-si, Gyeongsangbuk-do, Republic of Korea

**Keywords:** foot-and-mouth disease, vaccine, adjuvant, isoprinosine, innate and adaptive immunity, host defense

## Abstract

**Background:**

Commercial foot-and-mouth disease (FMD) vaccines have limitations, such as local side effects, periodic vaccinations, and weak host defenses. To overcome these limitations, we developed a novel FMD vaccine by combining an inactivated FMD viral antigen with the small molecule isoprinosine, which served as an adjuvant (immunomodulator).

**Method:**

We evaluated the innate and adaptive immune responses elicited by the novel FMD vaccine involved both in vitro and in vivo using mice and pigs.

**Results:**

We demonstrated isoprinosine-mediated early, mid-term, and long-term immunity through in vitro and in vivo studies and complete host defense against FMD virus (FMDV) infection through challenge experiments in mice and pigs. We also elucidated that isoprinosine induces innate and adaptive (cellular and humoral) immunity via promoting the expression of immunoregulatory gene such as pattern recognition receptors [PRRs; retinoic acid-inducible gene (RIG)-I and toll like receptor (TLR)9], transcription factors [T-box transcription factor (TBX)21, eomesodermin (EOMES), and nuclear factor kappa B (NF-kB)], cytokines [interleukin (IL)-12p40, IL-23p19, IL-23R, and IL-17A)], and immune cell core receptors [cluster of differentiation (CD)80, CD86, CD28, CD19, CD21, and CD81] in pigs.

**Conclusion:**

These findings present an attractive strategy for constructing novel FMD vaccines and other difficult-to-control livestock virus vaccine formulations based on isoprinosine induced immunomodulatory functions.

## 1 Introduction

Foot-and-mouth disease (FMD) is an acute infectious viral disease that affects cloven-hoofed animals, including cattle, buffaloes, pigs, sheep, and goats (Arzt et al., 2011a, b; Hwang et al., 2021). Typical symptoms of FMD include a high fever lasting 2–3 days and the development of blisters in the mouth and feet. The FMD virus (FMDV) can spread through various means, including close contact, animal-to-animal transmission, aerosol transmission, and vectors (Paton et al., 2018). Due to the significant damage it causes to the livestock industry, characterized by rapid virus replication, extensive virus spread, and mortality due to myocarditis in young animals, many countries have established strategies to prevent FMD, with vaccination being a common approach.

However, currently available commercial vaccines for FMD have certain drawbacks. These include local side effects at the injection site caused by mineral oil emulsions, such as ISA 206, ISA 50, and Marcol 52, the need for periodic vaccinations due to short-lived antibody titers, and incomplete host defense primarily relying on humoral immunity. To address these limitations, the use of a potent immunostimulatory adjuvant has been proposed. An adjuvant is any substance that enhances or prolongs the immune response when combined with a specific vaccine antigen (Cao et al., 2020). Furthermore, adjuvants amplify the immune response to a vaccine with a limited antigen content and regulate the host’s immune response (Zhao et al., 2023).

In this study, we developed an FMD vaccine incorporating the small molecule isoprinosine as an immunomodulatory adjuvant to overcome the shortcomings of existing FMD vaccines. Isoprinosine, an antiviral drug, combines inosine and dimepiranol acedoben in a 1:3 ratio and functions as an immunostimulant. As a vaccine adjuvant, isoprinosine promotes vaccine-induced type 1 helper T cell (Th1 cell)-mediated immune responses by stimulating the secretion of proinflammatory cytokines, including interferon (IFN)γ and interleukin (IL)-2. Th1 cells play a pivotal role in T-lymphocyte maturation and differentiation, thereby enhancing the induced lymphocyte proliferative response (Tsang et al., 1985; Milano et al., 1991; Petrova et al., 2010; Lasek et al., 2015). Activated Th1 cells also promote B lymphocyte differentiation, leading to the induction of humoral immunity (Tsang et al., 1987). Consequently, isoprinosine can elicit innate and adaptive immunity by modulating the secretion of these cytokines. In terms of innate immunity, isoprinosine induces neutrophil, monocyte, and macrophage (MΦ) phagocytosis and chemotaxis, as well as natural killer (NK) activity in eosinophils, through increased levels of immunoglobulin (Ig)G and complement surface markers (Ohnishi et al., 1983; Tsang et al., 1983). Additionally, isoprinosine has been observed to stimulate host-cell RNA synthesis while inhibiting viral RNA synthesis (Gordon and Brown, 1972; Ohnishi et al., 1982). Isoprinosine can also restore weakened T lymphocyte function by increasing cellular ribosomal RNA and protein synthesis and interfering with viral replication (Sliva et al., 2019). These properties led us to employ isoprinosine as an adjuvant in a novel FMD vaccine, aiming to induce long-term immunity, comprehensive host defense, concurrent cellular and humoral immunity, and modulating innate immunity.

In this study, we conducted *in vitro* and *in vivo* investigations in mice and pigs to assess the efficacy of our novel FMD vaccine containing isoprinosine. Our findings demonstrate that isoprinosine can elicit long-term immunity and comprehensive host defense. Additionally, our study provides evidence of isoprinosine’s pivotal role in combating FMD and reveals an uncharted immunological mechanism initiated by isoprinosine in pigs.

## 2 Materials and methods

### 2.1 Antigen purification

Purified antigens were prepared from BHK-21 (baby hamster kidney-21; ATCC, Manassas, VA, USA) cells infected with type O-serotype FMDV O PA2 (O/PAK/44, GenBank Accession No. AY593829.1) or type A-serotype FMDV A YC (A/SKR/YC/2017, GenBank Accession No. KY766148.1), following a modified method described by Lee et al (Lee et al., 2022; Kim et al., 2023). In brief, cytopathic effect (CPE) was observed 16 h after FMDV infection, and the virus was inactivated by treatment with 0.003 N binary ethylenimine (BEI) twice for 24 h. Once virus inactivation was confirmed via an inactivation confirmation test, it was concentrated using polyethylene glycol (PEG) 6000 (Sigma-Aldrich, St. Louis, MO, USA) (Bahnemann, 1975; Lee et al., 2022). The concentrated virus was then layered on a 15–45% sucrose density gradient and subjected to ultracentrifugation (30,000 rpm, 4h, 4°C, SW 41Ti, Beckman Coulter). To ensure virus inactivation, the BEI-treated supernatant was passed through ZZ-R 127 (fetal goat tongue; ATCC) and BHK-21 cells at least twice to verify the absence of any cytopathic effect (Kim et al., 2023).

### 2.2 Animals and ethics statement

The management of mice (experimental animals) and pigs (target animals) was conducted as previously described (Lee et al., 2022; Kim et al., 2023). Health- and age-matched C57BL/6 mice (females; 6–7 weeks-old) were obtained from KOSA BIO Inc. (Gyeonggi-do, Korea) for the fundamental experiments. Farm pigs (8–9 weeks-old), which tested negative for FMDV type O and A antibodies in their blood, were used for the target animal experiments. Throughout the study, all animals were housed in a specific pathogen-free (SPF) animal biosafety level 3 (ABSL3) facility in accordance with the Animal and Plant Quarantine Agency (APQA) guidelines. The housing room maintained conditions at 22°C, humidity at 50%, and a 12-h light/dark cycle. The study was approved by the Ethics Committee of the APQA (certification no.: IACUC-2022-658).

### 2.3 Cell viability assay

BHK-21, LF-BK (fetal porcine kidney; supplied by Plum Island Animal Disease Center, Orient, NY, USA), ZZ-R cells (2 × 10^4^ cells/well), murine peritoneal exudate cells (PECs), and porcine peripheral blood mononuclear cells (PBMCs) (1 × 10^5^ cells/well) were cultured in a 96-well plate for 48 h. Then, isoprinosine (Cayman, Ann Arbor, MI, USA; 0, 0.625, 1.25, 2.5, or 5 μg/mL) was added and cultured for 4 h. Cell viability was assessed using an MTS assay (CellTiter 96 AQueous One Solution Reagent; Promega, Madison, WI, USA). Data were obtained using a Hidex 300SL spectrophotometer (Hidex, Turku, Finland) at 490 nm.

### 2.4 Murine PECs isolation

Murine PECs were isolated from naive mice as previously described (Lee et al., 2022; Kim et al., 2023). Naive mice were anesthetized with CO_2_ and euthanized. The abdominal cavity was flushed with 5 mL of Ca^2+^/Mg^2+^-free Dulbecco’s PBS (Gibco, Waltham, MA, USA). The harvested peritoneal lavage fluid was centrifuged (300 g, 10 min, 4°C) and the pelleted PECs were resuspended and counted. Cryopreserved PECs were not used in any experiments.

### 2.5 Porcine PBMCs isolation

Porcine PBMCs were isolated from the whole blood of pigs (8–9 weeks-old animals, *n* = 3/group) as previously described (Lee et al., 2022; Kim et al., 2023). Whole blood (10 mL/donor) was individually collected and PBMCs were isolated using Histopaque (Sigma-Aldrich). PBMCs were suspended in Ca^2+^/Mg^2+^-free Dulbecco’s PBS (Gibco) and counted. Purified PBMCs were incubated in RPMI 1640 (Gibco) medium. All cells were freshly isolated before use.

### 2.6 IFNγ secretion induced by inactivated FMDV antigen with or without isoprinosine using the ELISpot assay *in vitro* in murine PECs and porcine PBMCs

Isoprinosine with or without antigen-induced IFNγ secretion was analyzed using a commercial ELISpot assay kit (R&D Systems, Minneapolis, MN, USA) as per manufacturer’s instructions. Murine PECs (5 × 10^5^ cells/well) or porcine PBMCs (5 × 10^5^ cells/well) were stimulated with 2 μg/mL (final concentration) of inactivated FMDV O PA2 or A YC antigen mixed with 0, 0.625, 1.25, 2.5, and 5 μg/mL of isoprinosine sequentially for 18 h in a humidified incubator at 37°C with 5% CO_2_. PBS and antigen (O PA2 and A YC) were used as negative control (NC) and positive control (PC), respectively. Data were obtained using the ImmunoSpot ELISpot reader (Autoimmune Diagnostika GmbH, Strassberg, Germany).

### 2.7 Early host defense in experimental animals (mice) administered with isoprinosine alone

To evaluate isoprinosine alone-mediated host defense against FMDV, the experimental (Exp) group mice received 100 μg isoprinosine/dose/mouse for a total volume of 100 μL. Mice in the NC group were administered an equal volume of PBS (pH 7.0) via the same route. Mice (6–7 weeks old, n = 5/group) were administered by intramuscular (I.M.) injection in the thigh muscle 0 days post-injection (dpi) and challenged with FMDV (100 LD_50_ of O/VET/2013 or 100 LD_50_ A/Malay/97) by intraperitoneal (I.P.) injection at 3 dpi or 7 dpi. Survival rates and body weight were monitored up to 7 days post-challenge (dpc) to evaluate host defense effectiveness.

### 2.8 Preparation of test vaccine

Inactivated antigens (O PA2 and A YC) were mixed (adjuvanted) with 10% Al(OH)_3_ in an ice and left to stand for 1 hr to adsorb and create a depot. After dispensing Quil-A and isoprinosine into the suspension (aqueous phase) containing antigen and 10% Al(OH)_3_, the weight of the aqueous layer was adjusted with TK buffer (Tris-KCl; pH 7.4). After dispensing ISA206 (oil-based emulsion, 50%, w/w), homogenization was performed at low speed (1200 rpm) with ultrahomogenizer in an ice bath according to the manufacturer’s instructions. Emulsification was continued until a milky, low viscosity and stable emulsion was obtained. The prepared vaccine was stored at 4°C until vaccination to animals and regularly monitored for stability and immunogenicity.

### 2.9 FMDV challenge after immunization with the FMD vaccine containing isoprinosine in mice

The animal experiments were conducted as previously described (Lee et al., 2022; Kim et al., 2023). We performed animal experiments to evaluate the potential of isoprinosine to serve as a vaccine adjuvant and validate the protective effects mediated by the FMD vaccine containing isoprinosine in the early stages of viral infection. The vaccine composition used in the experiments for the PC group was as follows: purified antigens (O PA2 and A YC) (0.375 + 0.375 μg/dose/100 μL), ISA 206 (Seppic, Paris, France; 50% w/w), 10% Al(OH)_3_, and 15 μg/dose/mouse Quil-A (InvivoGen, San Diego, CA, USA), in a total volume of 100 μL. The Exp group received vaccines with the same formula as the PC group, adding 100 μg isoprinosine/dose/mouse as an adjuvant via the same route. Mice in the NC group were administered an equal volume of PBS (pH 7.0) via the same route. Mice (6–7 weeks old, *n* = 5/group) were vaccinated by means of an I.M. injection [0 days post-vaccination (dpv)] and challenged with FMDV (100 LD_50_ of O/VET/2013 or 100 LD_50_ A/Malay/97) by means of an I.P. injection at 7 dpv. Survival rates and body weight were monitored up to 7 dpc (Lee et al., 2022; Kim et al., 2023).

### 2.10 Early, mid-term, and long-term immune response of FMD vaccines containing isoprinosine as an adjuvant in mice

To evaluate the efficacy of isoprinosine in eliciting early, mid-term, and long-term immune responses as an FMD vaccine adjuvant, we prepared an FMD vaccine that included isoprinosine as an adjuvant, especially as an immunostimulant, following the protocol described by Lee et al. and Kim et al (Lee et al., 2022; Kim et al., 2023). Mice (6–7 weeks old, *n* = 5/group) were immunized with the test vaccine via the I.M. route, and blood samples were collected at 0, 7, 28, 56, and 84 dpv for serological analysis, including structural protein O and A ELISAs and virus neutralization (VN) test for O PA2 and A.

### 2.11 Early, mid-term, and long-term immune response of FMD vaccines containing isoprinosine as an adjuvant in pigs

To evaluate the efficacy of isoprinosine in inducing early, mid-term, and long-term immune responses as an FMD vaccine adjuvant in target animals, experiments were performed as previously described (Lee et al., 2022; Kim et al., 2023).

Pigs were randomly divided into three groups: NC, PC, and Exp group (8–9 weeks old, *n* = 5–6/group). The vaccine composition used in the experiments for the PC group was as follows: purified antigens (O PA2 and A YC) (15 + 15 μg/dose/mL), ISA 206 (50% w/w), 10% Al(OH)_3_, and 150 μg/dose/pig Quil-A (InvivoGen), in a total volume of 1 mL. The Exp group received vaccines with the same formula as the PC group, adding 1 mg isoprinosine/dose/pig as an adjuvant via the same route. Pigs in the NC group were administered an equal volume of PBS (pH 7.0) via the same route. After primary vaccination via an I.M. injection, a booster shot was injected at 28 dpv via the same route. Blood was collected at 0, 7, 14, 28, 42, 56, and 84 dpv for serological assays, such as SP O and SP A ELISAs, VN test, and isotype-specific antibody immunoassay.

### 2.12 Serological assays

To detect SP antibodies in serum samples, the VDPro^®^ FMDV type O kit (Median Diagnostics, Gangwon-do, Korea) and PrioCheck™ FMDV type A kit (Prionics AG, Schlieren, Switzerland) were used according to the manufacturer’s guidelines. Mice and pigs had both FMDV type O- and FMDV type A-specific antibodies; consequently, the types of FMDV antigen coated on the VDPro^®^ kit and PrioCheck™ kit were different. Therefore, we measured the antibody titer using both kits to prevent underestimation by the characteristics of the antigen coating the kit. Absorbance was assessed within 30 min using spectrophotometer (Hidex) at 450 nm. The absorbance in the ELISA plate was converted to the percent inhibition (PI) value. The animals were considered antibody-positive when the PI value was ≥ 40% for the VDPro^®^ FMDV kit or ≥ 50% for the PrioCheck™ FMDV kit.

The VN test was conducted as per World Organization for Animal Health (WOAH) manual (Fowler et al., 2010). Sera samples were heat-inactivated, diluted, and incubated with a 100 TCID_50_ (50% tissue culture infective dose) in 50 μL media of FMDV (O PA2 or A YC) at 37°C for 1 h. Subsequently, 50 μL of LF-BK cells (10^6^ cells/mL) were added to each well and incubated at 37°C in a 5% CO_2_ atmosphere for 3 days, and the wells were checked for CPE. Antibody titers were evaluated as the Log_10_ of the reciprocal antibody dilution required for neutralization of 100 TCID_50_ of viruses in 50% of the wells (Fukai et al., 2015).

To detect isotype-specific antibodies, ELISAs for porcine IgG, IgA, and IgM (Bethyl Laboratories Inc., Montgomery, TX, USA) were conducted on the serum samples, as per manufacturer’s instructions. Data were obtained using spectrophotometer (Hidex) at 450 nm.

### 2.13 RNA isolation, cDNA synthesis, and qRT-PCR

To investigate the mechanism of the immune response induced by the FMD vaccine with isoprinosine, an experiment was conducted as per previously described protocol (Lee et al., 2020, 2022; Kim et al., 2023). Porcine PBMCs derived total RNA was extracted using TRIzol reagent (Invitrogen) and RNeasy Mini Kit (QIAGEN, Valencia, CA, USA) as per manufacturer’s instructions. Complementary DNA (cDNA) was prepared by reverse transcription using the GoScript Reverse Transcription System (Promega) as per manufacturer’s instructions. The synthesized cDNAs were amplified by quantitative real-time PCR (qRT-PCR) on a Bio-Rad iCycler using iQ SYBR Green Supermix (Bio-Rad Laboratories, Hercules, CA, USA). Gene expression levels were normalized to HPRT (reference gene) levels and presented as relative ratios compared with the control. The primer list is described in **Supplementary Table 1**.

### 2.14 Host defense against FMDV infection in pigs, in response to FMD vaccines containing isoprinosine as an adjuvant

To assess the host defense against viral infection of FMD vaccines containing isoprinosine in target animals, challenge experiments were performed as previously described (Jo et al., 2021; Kim et al., 2023). The study was carried out in an ABSL3 facility at APQA, according to the aforementioned conditions and institutional guidelines.

The Exp group was immunized via I.M. injection with a single dose of FMD vaccine containing isoprinosine to evaluate host defense against FMDV infection. The composition of the test vaccine containing isoprinosine as an adjuvant was as follows: purified antigen (O PA2 and A YC) (15 + 15 μg/dose/pig/mL), with isoprinosine (1 mg/dose/pig/mL), ISA 206 (50% w/w), 10% Al(OH)_3_, and 150 μg/dose/pig Quil-A. The test vaccine was prepared as a single dose (1 mL) and vaccinated into the animals via an I.M. injection. The control groups (PC and NC) of pigs were treated with an equal volume of a commercial FMD vaccine (O Primorsky+A Zabaikalski, ARRIAH-VAC^®^ by FGBI “ARRIAH”) and PBS, respectively, via the same route. After vaccination at 0 dpv, serum samples were collected at 0 and 28 dpv to observe the antibody titers using SP O and A ELISAs and a VN test. Clinical signs and appetite were monitored daily.

The vaccinated pigs were challenged with FMDV type O (O/SKR/JC/2014, Asia topotype) at a dose of 10^5^ TCID_50_/100 μL via an intradermal injection on the heel bulb. Clinical signs were monitored daily from 0 to 8 dpc. Oral swab samples were collected daily from 0 to 8 dpc, using a BD™ Universal Viral Transport Kit (BD Biosciences, San Jose, CA, USA). Serum samples were collected at 0, 2, 4, 6, and 8 dpc through venipuncture (anterior vena cava).

Clinical scores were determined as described by Jo et al. (Jo et al., 2021) and Alves et al (Alves et al., 2009): (a) hoof and foot vesicles (1–2 points per foot) and (b) snout, lips, and tongue vesicles (1 point for each area).

For the viral load assay, viral RNA was extracted from sera and oral swab samples using the QIAcube^®^ HT Pathogen Kit (QIAGEN, Leipzig, Germany), and RT-PCR was performed using a one-step prime-script RT-PCR kit (Bioneer Inc., Daejeon, Republic of Korea). Data were obtained with a CFX96 TouchTM real-time PCR detection system (Bio-Rad).

### 2.15 Statistical analysis

All quantitative data have been expressed as the mean ± SEM unless otherwise stated. Between-group statistical differences were assessed using a two-way or one-way analysis of variance, followed by Tukey’s or Dunnett’s *post-hoc* test. Statistical significance was denoted as ^∗^ *p<*0.05, ^∗∗^ *p<*0.01, ^∗∗∗^ *p<*0.001, and ^∗∗∗∗^ *p*<0.0001. Parametric tests were used to compare the different groups. Survival curves were constructed using the Kaplan–Meier method, and differences were analyzed using the log-rank sum test. Prism 10.0.2 (GraphPad, San Diego, CA, USA) was used for all statistical analyses.

## 3 Results

### 3.1 Isoprinosine induces potent innate and cellular immunity through the expression of IFNγ and exhibits an adjuvant effect when combined with an inactivated FMDV antigen *in vitro*

Before assessing isoprinosine-induced IFNγ secretion, we confirmed the absence of cytotoxicity due to isoprinosine treatment in isolated murine (PECs, porcine PBMCs, as well as BHK-21, LF-BK, and ZZ-R cells, all susceptible to FMDV. As a result, we found no cytotoxic effects of isoprinosine at concentrations ranging from 0 to 5 μg/mL in these cell types (**Supplementary Figures 1A–E**).

**Figure 1:**
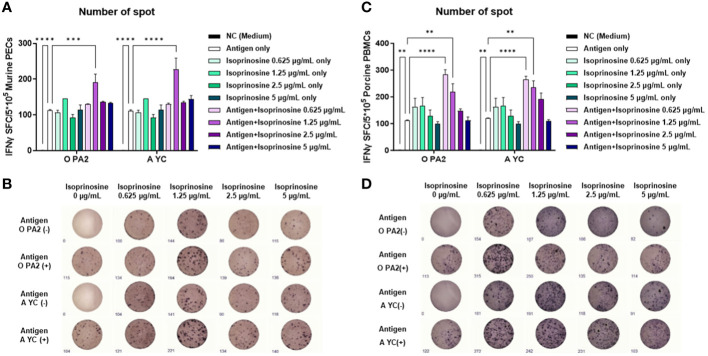
Innate and cellular immune response induced upon treatment with isoprinosine in combination with FMDV type O (O PA2) or A (A YC) antigen, in murine PECs and porcine PBMCs. IFNγ secretion mediated by O PA2 or A YC antigen, with or without isoprinosine, was assayed to evaluate the cellular immune responses induced by isoprinosine with or without FMDV antigen, using ELISpot assay, in murine PECs and porcine PBMCs. Data have been presented as spot-forming cells per number of cells in the well, and mean ± SEM of triplicate measurements (*n* = 3/group). **(A–D)** IFNγ-secreting cell spots in murine PECs **(A)**; Images of IFNγ secretion in murine PECs **(B)**; IFNγ-secreting cell spots in porcine PBMCs **(C)**; and Images of IFNγ secretion in porcine PBMCs **(D)**. Statistical analyses were performed using one-way ANOVA followed by Tukey’s *post-hoc* test. ^**^ *p* < 0.01; ^***^ *p* < 0.001; and ^****^ *p* < 0.0001.

To evaluate the innate and cellular immune responses triggered by inactivated FMDV antigens with or without isoprinosine, we conducted an ELISpot assay to measure the IFNγ production index (number of spots) (Ibrahim Eel et al., 2015). In the *in vitro* ELISpot assay using PECs, the Exp group containing isoprinosine showed a significantly higher IFNγ secretion index compared to the PC group administered either antigen (O and A) or isoprinosine alone (**Figures 1A, B**). Similarly, in the *in vitro* ELISpot assay using PBMCs, the Exp group containing isoprinosine also exhibited a significantly higher IFNγ production index than the PC group administered either antigen (O and A) or isoprinosine alone (**Figures 1C, D**). These results highlight the potential of isoprinosine as a new FMD vaccine adjuvant capable of enhancing both innate and cellular immune responses, including IFNγ secretion, in mice and pigs.

### 3.2 The FMD vaccine containing isoprinosine as an adjuvant protects the host during the early stages of FMDV infection in mice

Before assessing the efficacy of isoprinosine as a vaccine adjuvant, we evaluated the protective effect of the host against viral infection when administered isoprinosine alone. Notably, all mice in the group administered isoprinosine alone succumbed to infection when challenged with FMDV type O [O/VET/2013 (ME-SA topotype)] and A [A/Malay/97 (SEA topotype)] at 3 and 7 days dpi, respectively. This confirmed that isoprinosine alone, without the FMDV antigen, did not confer host defense (**Supplementary Figures 2A–I**).

**Figure 2:**
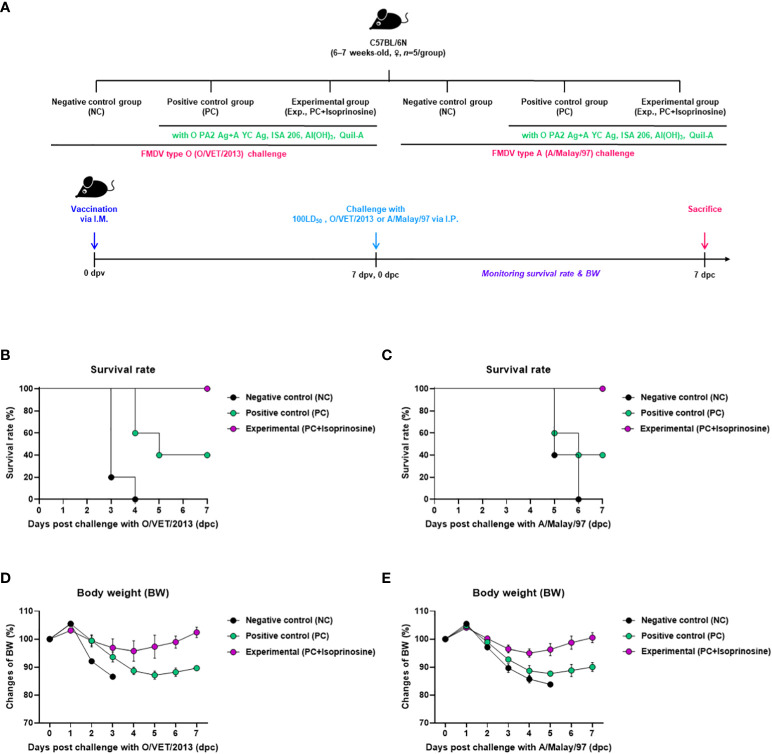
FMD vaccine containing isoprinosine enhances vaccine efficacy and protect the host in mice. C57BL/6 mice (6–7 weeks-old, *n* = 5/group) were administered an FMD vaccine containing inactivated FMDV type O (O PA2) and A (A YC) antigens (0.375 + 0.375 μg/dose/100 μL; 1/40 of the dose for pigs), 100 μg isoprinosine/dose/mouse, ISA 206 (50%, w/w), 10% Al(OH)_3_, and 15 μg Quil-A. The PC group received vaccines of the same volume and composition as the Exp group but without isoprinosine as an adjuvant. The NC group was injected with an equal volume of PBS. The test vaccines were injected via the intramuscular route into mice that were later challenged with FMDV O (100 Lethal Dose 50%, LD_50_ O/VET/2013) or FMDV A (100 LD_50_ A/Malay/97), at 7 dpv via the intraperitoneal route. Survival rates and body weights were monitored for 7 dpc. **(A–E)** experimental strategy **(A)**; survival rates post-challenge with O/VET/2013 **(B)** and A/Malay/97 **(C)**; and changes in body weight post-challenge with O/VET/2013 **(D)** and A/Malay/97 **(E)**. Data have been represented as the mean ± SEM of triplicate measurements (*n*=5/group).

To investigate the adjuvanticity of isoprinosine and the initial host defense provided by the FMD vaccine containing isoprinosine against viral infection, we conducted the following experiments, as illustrated in **Figure 2A**. A bivalent vaccine containing isoprinosine along with O PA2+A YC antigens exhibited 100% survival rates against FMDV type O and A (**Figures 2B, C**) (Lee et al., 2022; Kim et al., 2023). Body weight changes in the mice from the Exp group administered isoprinosine remained minimal (**Figures 2D, E**). In contrast, the PC group, which was not administered isoprinosine, showed survival rates of 60% and 40% for FMDV type O and A infections, respectively, with a bodyweight decrease exceeding 10% at 4 dpc (Jo et al., 2021). The NC group had a 100% mortality rate (0% survival) at 4 dpc and 6 dpc for FMDV type O and A challenges, respectively. These results prove that the novel FMD vaccine containing isoprinosine successfully establishes initial host defense in mice.

### 3.3 FMD vaccine containing isoprinosine as an adjuvant elicits potent humoral immunity in mice and pigs

**Figure 3:**
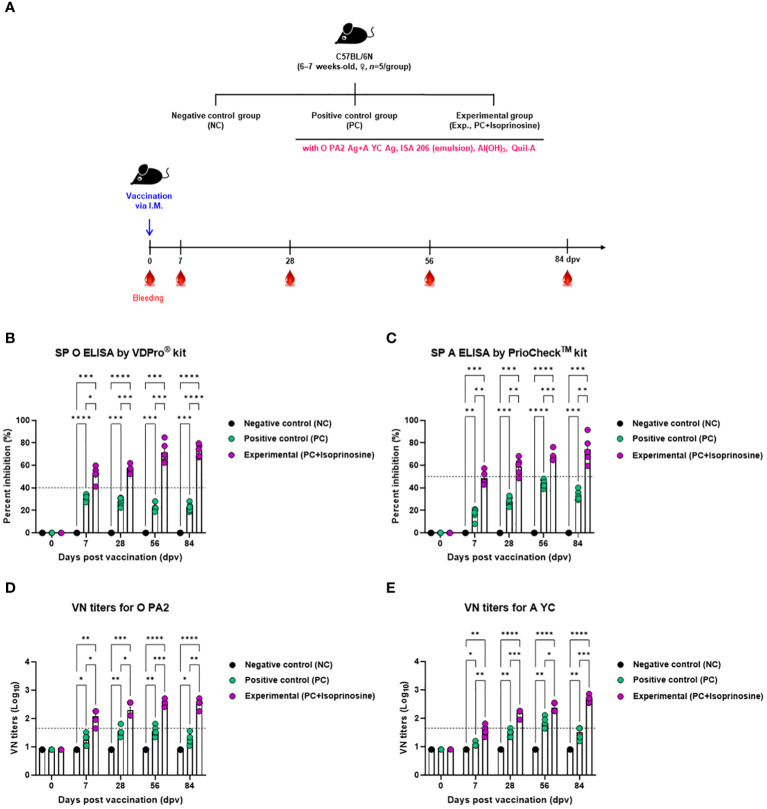
FMD vaccine containing isoprinosine elicits potent humoral immune response in mice. C57BL/6 mice (6–7 weeks-old, *n* = 5/group) were administered an FMD vaccine containing inactivated FMDV type O (O PA2) and A (A YC) antigens (0.375 + 0.375 μg/dose/100 μL; 1/40 of the dose for pigs), 100 μg isoprinosine/dose/mouse, ISA 206 (oil-based emulsion, 50%, w/w), 10% Al(OH)_3_, and 15 μg Quil-A. The PC group received vaccines of the same volume and composition as the Exp group but without isoprinosine as an adjuvant. The NC group was injected with an equal volume of PBS. Mice were vaccinated with the test vaccine via the intramuscular route, and blood was collected at 0, 7, 28, 56, and 84 dpv, for serological analysis using SP O and A ELISAs and VN titers for O/PKA/44/2008 (O PA2) and A/SKR/YC/2017 (A YC). **(A–E)** experimental strategy **(A)**; Ab titers, as determined using SP O **(B)** and SP A **(C)** ELISAs; and VN titers for O PA2 **(D)** or A YC **(E)**, as determined using VN test. Data are represented as mean ± SEM of triplicate measurements (*n* = 5/group). Statistical analyses were performed using two-way ANOVA followed by Tukey’s *post-hoc* test. ^*^ *p* < 0.05; ^**^ *p* < 0.01; ^***^ *p* < 0.001; and ^****^ *p* < 0.0001.

To assess the humoral immune response induced by the FMD vaccine using isoprinosine as an adjuvant, we examined early, mid-term, and long-term immune responses in mice (**Figure 3A**). We monitored antibody titers using SP O ELISA and VN titers using a VN test (**Figures 3B-E**).

Upon administration of the FMD vaccine with isoprinosine to mice in the Exp group, antibody titers at 7 (*p* < 0.05, SP O ELISA using the VDPro^®^ Kit; *p* < 0.01, SP A ELISA using the PrioCheck™ Kit), 28 (*p* < 0.001, O PA2; *p* < 0.01, A YC), 56 (*p* < 0.001, both O PA2 and A YC), and 84 (*p* < 0.0001, O PA2; *p* < 0.01, A YC) days post-vaccination (dpv) were significantly higher than those in the PC group (**Figures 3B, C**).

VN titers against FMDV (O PA2 and A YC) were also higher in the Exp group treated with the vaccine containing isoprinosine along with the O PA2+A YC antigens at 7 (*p* < 0.05, O PA2; *p* < 0.01, A YC), 28 (*p* < 0.05, O PA2; *p* < 0.001, A YC), 56 (*p* < 0.001, O PA2; *p* < 0.05, A YC), and 84 (*p* < 0.01, O PA2; *p* < 0.001, A YC) dpv compared to the PC group. Notably, the VN titers in the NC group showed no significant changes (**Figures 3D, E**).

**Figure 4:**
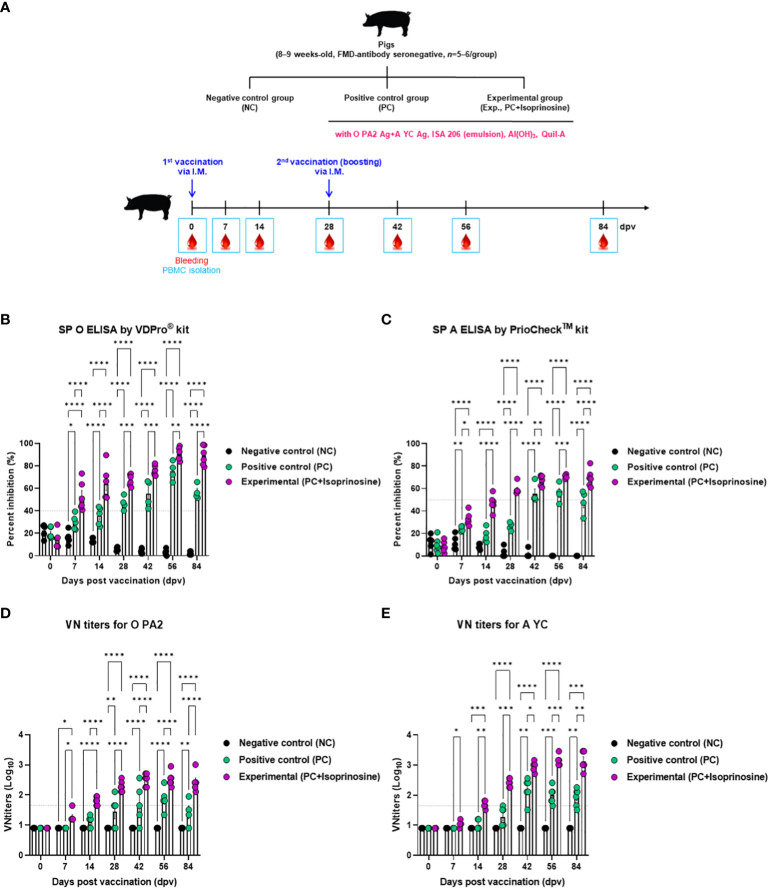
FMD vaccine containing isoprinosine elicits potent humoral immune response in pigs. For the pig experiments, FMDV type O and type A antibody-seronegative animals (8–9 weeks-old) were used. The pigs were divided into three groups (*n* = 5–6/group) and administered inactivated bivalent FMDV vaccine without (PC group) or with 1 mg/dose/pig isoprinosine (Exp group). The PC group received FMDV type O (O PA2) and type A (A YC) antigens (15 + 15 μg/dose/mL, one dose for cattle and pig use) with ISA 206 (50%, w/w), 10% Al(OH)_3_, and 150 μg Quil-A. Vaccination was performed twice at 28-day intervals, with 1 mL vaccine (one dose) injected via the deep intramuscular route into the necks of the animals. The NC group was injected with an equal volume of PBS. Blood samples were collected from pigs at 0, 7, 14, 28, 42, 56, and 84 dpv, for serological assays. **(A–E)** experimental strategy **(A)**; Abs titers, as determined using SP O **(B)** and SP A **(C)** ELISAs; and VN titers for O PA2 **(D)** or A YC **(E)**, as determined using VN test. Data have been represented as mean ± SEM of triplicate measurements (*n* = 5–6/group). Statistical analyses were performed using two-way ANOVA followed by Tukey’s *post-hoc* test. ^*^ *p* < 0.05; ^**^ *p* < 0.01; ^***^ *p* < 0.001; and ^****^ *p* < 0.0001.

Furthermore, targeted animal experiments were conducted to assess the humoral immune response induced by the FMD vaccine with isoprinosine as an adjuvant, using FMD antibody seronegative pigs (**Figure 4A**). The vaccines containing isoprinosine were administered to pigs to confirm early, mid-term, and long-term immune responses. The control groups (PC and NC) received the vaccine without isoprinosine and phosphate-buffered saline (PBS) through the same injection route.

Antibody titers specific to FMDV type O (SP O ELISA using the VDPro^®^ Kit) in the Exp group vaccinated with isoprinosine were higher than those in the PC group until 28 dpv. Following a booster shot at 28 dpv, the antibody titers in the Exp group continued to rise at 42 and 56 dpv, remaining higher than those in the PC group until 84 dpv (**Figure 4B**).

Antibody titers measured by SP A ELISA (using the PrioCheck™ Kit) increased somewhat more slowly compared to those determined using SP O ELISA. Nevertheless, at 7 dpv, the antibody titers in the Exp group were higher than those in the PC group, and seropositive levels persisted until 84 dpv. In the PC group, seronegative levels were maintained up to 28 dpv, with antibody titers reaching seropositive levels after the booster at 28 dpv. However, after 56 dpv, antibody titers tended to decrease and eventually reached seronegative levels at 84 dpv (**Figure 4C**).

**Figure 5:**
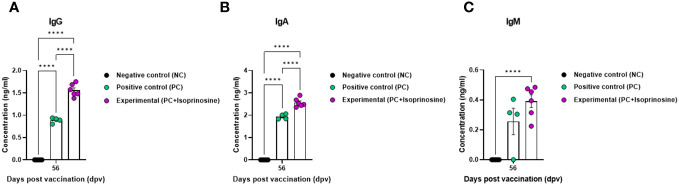
FMD vaccine containing isoprinosine induces an increase in the levels of the immunoglobulin subtypes IgG, IgA, and IgM, in pigs. For the pig experiments, FMDV type O and type A antibody-seronegative animals (8–9 weeks-old) were used. The experimental strategy and method were described in the legend in **Figure 4**. **(A–C)** IgG **(A)**; IgA **(B)**; and IgM **(C)** concentrations. Data are represented as the mean ± SEM of triplicate measurements (*n* = 5–6/group). Statistical analyses were performed using two-way ANOVA followed by Tukey’s *post-hoc* test. ^****^ *p* < 0.0001.

To measure the VN titer in the Exp group administered with isoprinosine and FMDV (O PA2+A YC) antigens, we used viruses homologous to O PA2 and A YC antigens (O/PKA/44/2008 and A/SKR/YC/2017, respectively) for comparison with the PC group. The VN titers increased from 7 to 84 dpv in the Exp group, surpassing those in the PC group (**Figures 4D, E**). To validate the effect of the FMD vaccine with or without isoprinosine on total IgG, IgA, and IgM levels in pigs, an isotype-specific ELISA was conducted using serum samples from pigs vaccinated at 56 dpv (**Figure 4A**). Levels of IgG and IgA were higher in the Exp group compared to the control group (PC and NC). There were no differences in IgM levels between the Exp and PC groups (**Figures 5A-C**). These results highlight that the FMD vaccine containing isoprinosine effectively elicits potent humoral immune responses.

### 3.4 FMD vaccine containing isoprinosine exhibits immunomodulatory functions by mediating the gene expression of pro-inflammatory cytokines, transcription factors, and cell core receptors

To elucidate the cellular and humoral immune response and mechanism induced by the FMD vaccine containing isoprinosine, we performed quantitative real-time PCR (qRT-PCR) using porcine PBMCs at specific time points (**Figure 4A**).

We observed changes in the gene expression of immunomodulatory molecules, including pattern-recognition receptors [PRRs; retinoic acid-inducible gene (*RIG*)-*I* and toll-like receptor (*TLR*)*9*], transcription factors [signal transducer and activator of transcription (*STAT*)*1*, *STAT4*, myeloid differentiation factor (*MYD*)*88*, T-box transcription factor (*TBX*)*21*, eomesodermin (*EOMES*), and nuclear factor kappa B (*NF-κB*)], cytokines (*IFNα*, *IFNβ*, *IFNγ*, *IL-1β*, *IL-6*, *IL-12p40*, *IL-23p19*, *IL-23R*, and *IL-17A*), and co-stimulatory molecules [cluster of differentiation (*CD*)*80*, *CD86*, *CD28*, *CD19*, *CD21*, and *CD81*] at 14 and 56 dpv.

**Figure 6:**
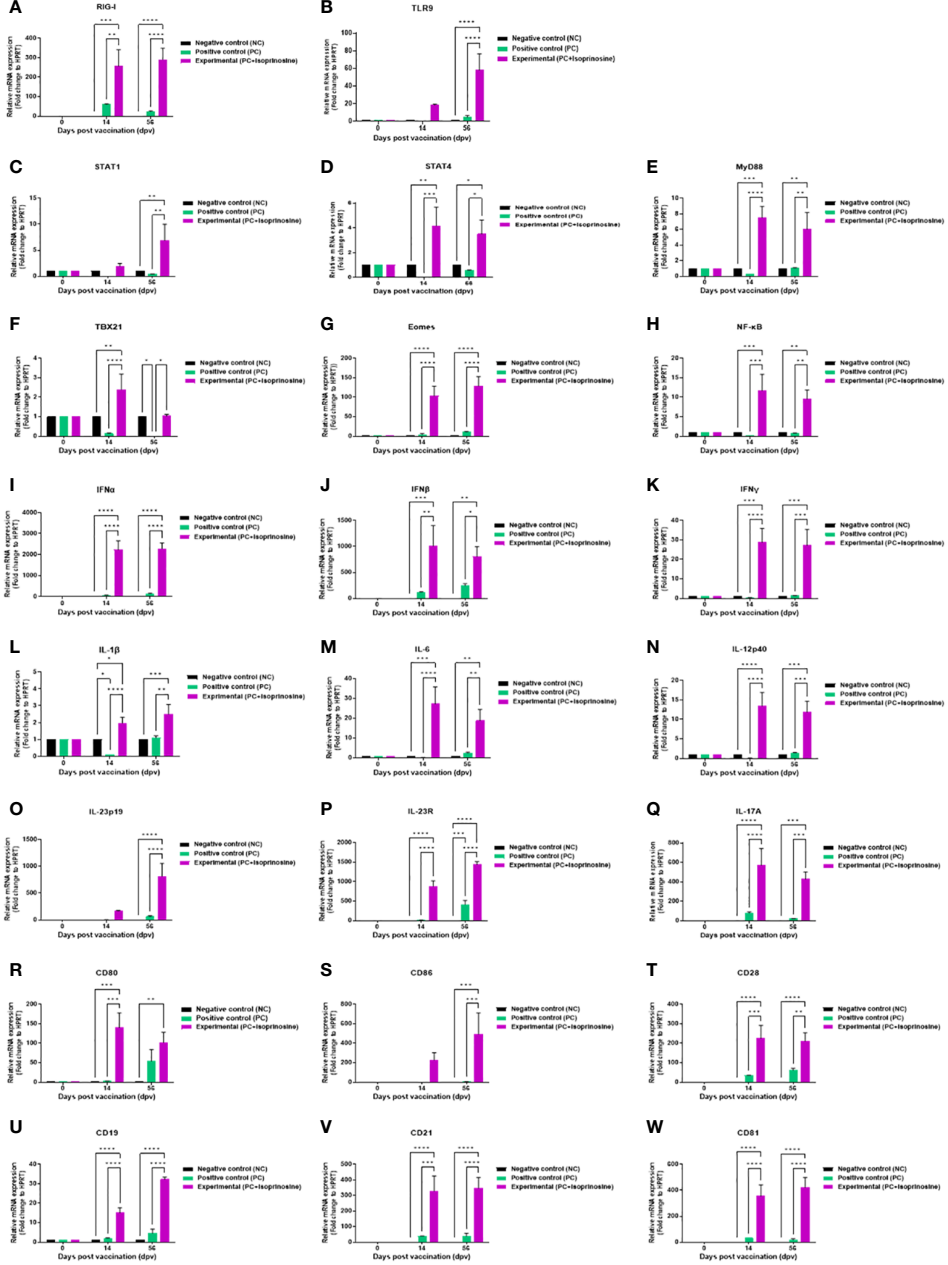
FMD vaccine containing isoprinosine induces the gene expression of PRRs, cytokines, co-stimulatory molecules, and transcription factors in porcine PBMCs. Porcine PBMCs isolated from the whole blood of vaccinated pigs (*n* = 5–6/group), as described in **Figure 4A**, were used for qRT-PCR. Gene expression levels were normalized to those of HPRT and have been presented as relative ratios compared to the control levels. **(A–W)** Gene expression levels of *RIG-I* **(A)**; *TLR9* **(B)**; *STAT1* **(C)**; *STAT4* **(D)**; *MYD88* **(E)**; *TBX21* **(F)**; *EOMES* **(G)**; *NF-κB* **(H)**; *IFNα* **(I)**; *IFNβ* **(J)**; *IFNγ* **(K)**; *IL-1β* **(L)**; *IL-6* **(M)**; *IL-12p40* **(N)**; *IL-23p19* **(O)**; *IL-23R* **(P)**; *IL-17A* **(Q)**; *CD80* **(R)**; *CD86* **(S)**; *CD28* **(T)**; *CD19* **(U)**; *CD21* **(V)**; and *CD81* **(W)**. Statistical analyses were performed using two-way ANOVA followed by Tukey’s *post-hoc* test. ^*^ *p* < 0.05; ^**^ *p* < 0.01; ^***^ *p* < 0.001; and ^****^ *p* < 0.0001.

PRRs *RIG-I* and *TLR9*, associated with transcription factors, exhibited elevated expression levels in the Exp group containing isoprinosine compared to the control groups (PC and NC). *RIG-I* expression was significantly upregulated in the Exp group at 14 and 56 dpv (**Figure 6A**). The level of *TLR9* was higher in the Exp group than in the control groups at 14 dpv, but the difference was not statistically significant. However, at 56 dpv, the levels in the Exp group were significantly higher than those in the control groups (PC and NC) (**Figure 6B**).

Expression levels of transcription factors were relatively lower than those of PRRs and cytokines but were upregulated in the Exp group compared to the control groups (PC and NC). *STAT1* levels were highest in the Exp group at 14 dpv, although the difference was statistically insignificant. However, at 56 dpv, *STAT1* levels in the Exp group were significantly increased compared to those in the control groups (PC and NC) (**Figure 6C**). The levels of *STAT4* (**Figure 6D**), *MYD88* (**Figure 6E**) were significantly elevated in the Exp group at both 14 and 56 dpv. *TBX21* levels were significantly upregulated in the Exp group at 14 dpv. At 56 dpv, *TBX21* levels in the Exp group were higher than in the PC group but not significantly different from those in the NC group (**Figure 6F**). The levels of *EOMES* (**Figure 6G**), and *NF-κB* (**Figure 6H**) were significantly elevated in the Exp group at both 14 and 56 dpv.

Pro-inflammatory cytokine-inducing factors exhibited significantly upregulated expression in pigs administered the vaccine containing isoprinosine. Levels of type I IFNs (*IFNα* and *IFNβ*) were induced in the Exp group compared to the control groups (PC and NC) at 14 and 56 dpv (**Figures 6I, J**). *IFNγ* levels, while slightly lower than those of type I IFNs, were significantly higher in the Exp group than in the other groups (PC and NC) at 14 and 56 dpv (**Figure 6K**). *IL-1β* expression was upregulated in the Exp group compared to the NC group at 14 dpv, although the difference was not statistically significant. In contrast, *IL-1β* expression in the Exp group was significantly higher than in the PC group. At 56 dpv, *IL-1β* expression was upregulated in the Exp group compared to the other groups (PC and NC) (**Figure 6L**). *IL-6* expression in the Exp group was significantly higher than in the other groups (PC and NC) at 14 and 56 dpv (**Figure 6M**). *IL-12p40* expression in the Exp group was significantly higher than in the other groups (PC and NC) at 14 and 56 dpv (**Figure 6N**). At 14 dpv, *IL-23p19* expression in the Exp group was higher than in the other groups, although the difference was not statistically significant; by 56 dpv, however, this difference became statistically significant (**Figure 6O**). *IL-23R* (**Figure 6P**) and *IL-17A* (**Figure 6Q**) expression levels were significantly increased in the Exp group compared to the control groups (PC and NC) at both 14 and 56 dpv.

In pigs vaccinated with isoprinosine, the total expression levels of co-stimulatory molecules were as high as those of cytokines. At 14 dpv, *CD80* expression in the Exp group was significantly higher than in the control groups (PC and NC). At 56 dpv, *CD80* levels in the Exp group were higher than those in the PC group, although the difference was not statistically significant; however, it was significantly higher than in the NC group (**Figure 6R**). At 14 dpv, *CD86* expression was higher in the Exp group than in the other groups (PC and NC), although there was no significant difference (**Figure 6S**). The expression levels of *CD28* (**Figure 6T**), *CD19* (**Figure 6U**), *CD21* (**Figure 6V**), and *CD81* (**Figure 6W**) were higher in the Exp group than in the control groups (PC and NC) at both 14 and 56 dpv.

### 3.5 FMD vaccines containing isoprinosine exhibit potent protective effects against FMDV infection in pigs

**Figure 7:**
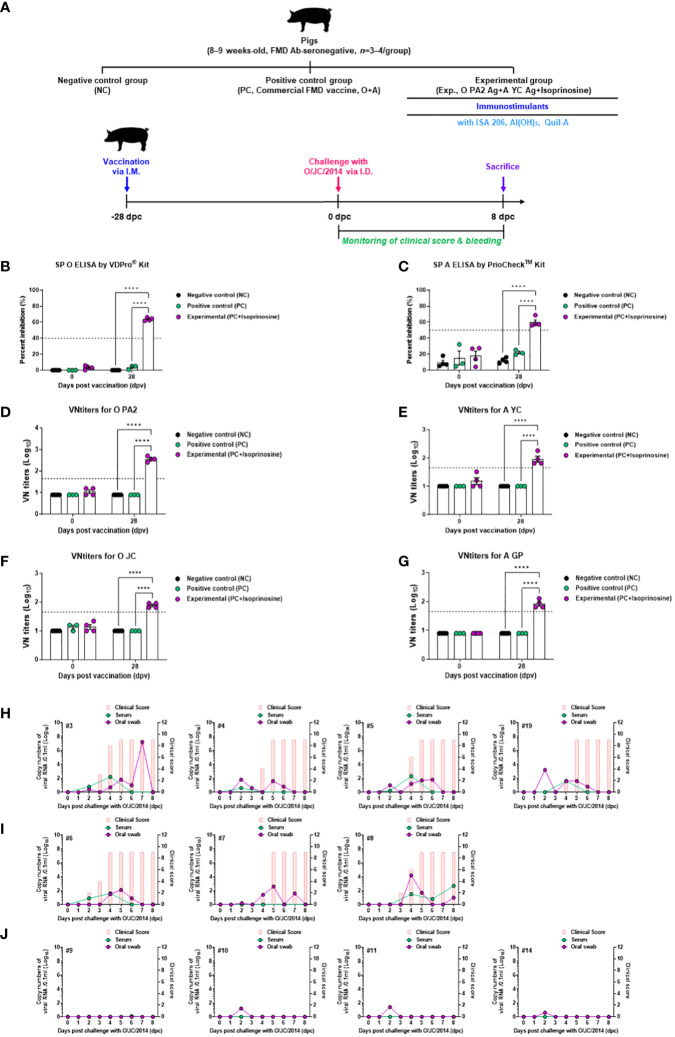
FMD vaccine containing Isoprinosine elicit potent host defense against FMDV infection in pigs. For the challenge experiments, FMDV type O and type A antibody-seronegative pigs (8–9 weeks-old, *n* = 3–4/group) were administered the FMD vaccine containing FMDV type O (O PA2) and type A (A YC) antigen (15 + 15 μg/dose/mL, one dose for cattle and pig use) with isoprinosine (1 mg/dose/pig), ISA 206 (50%, w/w), 10% Al(OH)_3_, and 150 μg Quil-A. One milliliter vaccine was prepared as a single dose and introduced into the animals, via an I.M. injection. The control groups (PC and NC) were treated with an equal volume of commercial FMD vaccine (O Primorsky+A Zabaikalski, ARRIAH-VAC^®^ by FGBI “ARRIAH”) and PBS, respectively, via the same route. Blood samples were collected at 0 and 28 dpv for serological assays. Vaccinated pigs were challenged with FMDV type O (O/SKR/JC/2014) on the heel bulb, at a dose of 10^5^ TCID_50_/100 μL, at 28 dpv. **(A–J)** experimental workflow **(A)**; Antibody titers, as assessed using SP O **(B)** and SP A **(C)** ELISAs; VN titers for O PA2 **(D)** or A YC **(E)** or O JC **(F)** or A GP **(G)**, as assessed using VN test; and Clinical score and viral load in serum samples and oral swabs from the NC group (*n* = 4/group) **(H)**, PC group (commercial FMD vaccine, *n* = 3/group) **(I)**, and Exp (O PA2+A YC+isoprinosine, *n* = 4/group) group **(J)** infected with FMDV type O (O/SKR/JC/2014). The left Y-axis of the graph shows the amount of virus in the serum and oral swab samples, represented as Log_10_ values, while the right Y-axis shows the clinical index as the maximum value of 10 points. Data are presented as the mean ± SEM of triplicate measurements (*n* = 3–4/group). Statistical analyses were performed using a two-way ANOVA followed by Tukey’s test. ^****^ *p* < 0.0001.

To validate the host defense provided by FMD vaccines containing isoprinosine in pigs, animals were vaccinated with a single dose (one shot) of the FMD vaccine containing isoprinosine as an adjuvant and challenged with FMDV type O (O/SKR/JC/2014, Asian topotype) at 28 dpv (**Figure 7A**). The SP O ELISA antibody titer measured using the VDPro^®^ Kits and PrioCheck™ Kits was increased in the Exp group containing isoprinosine compared to the control groups (PC and NC) (**Figure 7B**; **Supplementary Figure 3**). The SP A ELISA antibody titer measured using the PrioCheck™ Kit was also higher in the Exp group containing isoprinosine than in the other groups (PC and NC) (**Figure 7C**).

Upon challenges with O PA2 (**Figure 7D**), VN titers and A YC (**Figure 7E**) displayed similar time kinetics to the antibody titers. The VN titer for O/SKR/JC/2014 (O JC, **Figure 7F**) was higher in the Exp group than in the others (PC and NC). The VN titer upon challenge with A/SKR/GP/2018 (A GP, **Figure 7G**) increased in the Exp group compared to the control groups (PC and NC).

Several FMD parameters were analyzed after the challenge trials, including clinical signs, viremia in serum samples, and viral titers in oral swabs. NC groups (subjects #3, #4, #5, and #19) infected with FMDV type O (O/SKR/JC/2014) exhibited 100% (4/4) typical FMD clinical signs, with high levels of virus detected in the serum samples and oral swabs from these groups (**Figure 7H**). In the PC groups (subject numbers #6, #7, and #8), all individuals (3/3) exhibited clinical signs of FMD against FMDV type O infection, demonstrating a lack of host protection. Viremia in serum samples and viral shedding in oral swabs ranged from 1 to 3 (Log_10_) for FMDV type O (O/SKR/JC/2014) infection (**Figure 7I**). In contrast, the Exp groups (subject numbers #9, #10, #11, and #14) that received the vaccine containing isoprinosine did not show any clinical signs of FMD, and no virus secretion was found in their serum samples or oral swabs (**Figure 7J**).

These results prove that the FMD vaccine with isoprinosine as an adjuvant delivers complete host defense in pigs.

## 4 Discussion

FMDV is a positive-sense single-stranded RNA with a total length of approximately 8.3 kb (Logan et al., 2018). Seven serotypes of FMDV have been classified: O, A, Asia1, SAT1, SAT2, SAT3, and C (Grubman and Baxt, 2004). Because cross-protection between seven different serotypes is difficult, developing a novel FMD vaccine with an enhanced immune response with abroad spectrum of protective effects against various FMDV serotypes is urgent.

The effectiveness of the vaccine currently used for FMD could be improved by combining an adjuvant with the inactivated FMDV antigen. The adjuvants currently used in FMD vaccines include 1) Quil-A (Cox and Coulter, 1997), a water-extracted fraction of saponin that is less toxic and safer than crude saponin and has a superior ability to elicit cellular and humoral immune responses; and 2) aluminum hydroxide [Al(OH)_3_] (Park et al., 2014) and ISA 206 (Ibrahim Eel et al., 2015), which are the most frequently used adjuvants for inactivated FMD vaccines.

However, commercial FMD vaccine adjuvants have several limitations. FMD vaccines containing an oil adjuvant not only take a long time to induce antibody titers to the levels necessary to elicit host defense against viral infection but also have a short persistence of antibody titers. FMD vaccines containing oil adjuvants also cause local side effects at the injection site (Mahapatra and Parida, 2018; Lyons et al., 2019).

To address the disadvantages of an oil adjuvant, we developed a novel FMD vaccine containing inactivated antigens and the immunomodulator isoprinosine (Rweyemamu et al., 1989). Since isoprinosine was first approved in 1971, numerous studies have suggested that isoprinosine has beneficial effects on several diseases and infections (Sliva et al., 2019). Based on previous reports, isoprinosine has been demonstrated as a safe drug against serious side effects, such as cytotoxicity, genotoxicity, and mutagenicity (Tobólska et al., 2018a, b). The antiviral effect of isoprinosine has been demonstrated both *in vivo* and *in vitro* and is considered secondary to its immunomodulatory effects. Nevertheless, the exact mechanism of action of isoprinosine has not yet been elucidated (Sliva et al., 2019).

Isoprinosine-mediated cytotoxicity was confirmed before *in vitro* studies, but no cytotoxicity was observed at concentrations of 0–5 μg/mL (**Supplementary Figure 1**). Therefore, the following experiment was performed. We aimed to investigate FMDV antigen with or without isoprinosine-mediated innate immune response and cellular immune response by assessing the IFNγ secretion index (number of spots) using ELISpot assay. An ELISpot assay was performed using PECs isolated from mice and PBMCs isolated from pigs. PECs contain innate immune cells, including MΦs and dendritic cells (DCs). Unconventional T cells, including invariant NK, γδ, and mucosal-associated invariant (MAI) T cells, are also present in PECs (Jo et al., 2021). PBMCs consist of lymphocytes (NK, T, and B cells), monocytes, and DCs. Therefore, PECs and PBMCs are suitable for investigating cellular immune responses using ELISpot assays. The assay revealed that isoprinosine with or without viral antigen induced IFNγ secretion and demonstrated that even a low dose of isoprinosine (0.625 µg/mL) could induce IFNγ secretion (**Figure 1**).

We conducted mouse challenge experiments to demonstrate that the FMD vaccine with isoprinosine is capable of cross-protection and early host defense against FMDV serotypes O and A. The novel FMD vaccine containing isoprinosine exhibited a 100% survival rate, thereby highlighting the potential of isoprinosine as an FMD vaccine adjuvant (**Figure 2**). Host defense by administration of isoprinosine alone was not observed at 3 and 7 dpi (**Supplementary Figure 2**). However, considering the antiviral effect of isoprinosine, a protective effect against viral infection by administration of isoprinosine alone may have been observed if *in vivo* challenge experiments had been performed at an earlier time point (*e.g.* at 1 dpi).

To evaluate the effect of a bivalent vaccine containing isoprinosine on long-term immune responses in mice and pigs, SP ELISAs (types O and A) were performed, including the control groups (PC and NC). After boosting, both serotypes (types O and A) showed significant antibody titers in the Exp group, compared to those in the PC group, with little reduction until 84 dpv. These results provide evidence that isoprinosine induces long-term immunity in pigs by promoting memory responses and that the antibody titers generated can be maintained longer than 84 dpv (**Figures 3**, **4**).

To define the FMDV-neutralizing effect of the isoprinosine-induced long-term immune response, we performed a VN test using the same strategy as that used to evaluate antibody titers by means of SP ELISA. Vaccinated VN titers of >2 (Log_10_) were assessed to achieve host protection by the FMD vaccine (Black et al., 1984). In addition, according to Korea’s FMD vaccine efficacy validation guidelines, host defense was reportedly induced when the VN titer was >1.65 (Log_10_). After boosting, the Exp groups for both O and A types displayed significantly higher VN titers than the PC group. The Exp group also maintained a VN titer >2 (Log_10_) better than the PC group. The experimental results showed that the long-term immune response elicited by isoprinosine presents a possibility of resistance to FMDV infection at 84 dpv and beyond (**Figures 3**, **4**).

We also performed isotype ELISA at 56 dpv to identify the type of Ig generated by the isoprinosine-mediated immune response. The Exp group containing isoprinosine displayed a significant difference in IgG concentration compared to the PC group. Therefore, we demonstrated that isoprinosine significantly elicited a B cell-mediated humoral immune response compared to the PC group. Interestingly, despite I.M. vaccination, the Exp group displayed a dramatic difference in IgA concentration compared to the PC group (Fagarasan and Honjo, 2003). These results suggested that isoprinosine-induced T-box expressed in T cells (TBX21) enhances mucosal immunity, thereby presenting the possibility of oral vaccination (**Figures 5**, **6**) (Mohamed and Lord, 2016).

qRT-PCR was performed to determine the immunological mechanisms of isoprinosine-induced potent long-term immunity and host defense (**Figure 6**). Isoprinosine stimulates RIG-I and TLR9 on MΦs and DCs to promote the secretion of IFNα and IFNβ, which play crucial roles in the initial host defense. Activated MΦs and DCs contribute to the maturation and differentiation of neutrophils, Th17, and unconventional T cells (γδ, invariant NK, and MAIT cells) by inducing cytokines including IL-6 (Kimura and Kishimoto, 2010), IL-1β (Coffelt et al., 2015), IL-23p19 (Wang et al., 2019), and IL-17A (Ley et al., 2006).

Given the significant IL-17A secretion, isoprinosine stimulates and recruits neutrophils, releasing neutrophil extracellular traps (NETs). NETs are presumed to form in response to extracellular high mobility group box 1 (HMGB1) and histones in a TLR4- and TLR9-dependent manner and mice lacking these receptors show reduced liver pathology (Papayannopoulos, 2018). NETs trap and neutralize viruses, prevent their spread, and promote NET uptake via MΦ recruitment (Farrera and Fadeel, 2013). NETs also modulate other immune cells to orchestrate inflammatory cytokines directly or indirectly (Warnatsch et al., 2015). Isoprinosine induces IFNγ secretion by promoting TBX21 expression in Th1 and B cells through the EOMES, IL-12/STAT4, and IFNγ/STAT1 pathways. Th1 cells induce responses primarily triggered by MΦs and cytotoxic T cells (Belizário et al., 2016). They are induced by the pro-inflammatory cytokine IL-12 and the effector cytokines IFNγ and IL-2. The effector cells of immunity mediated by Th1 cells include MΦs, cytotoxic T cells, B cells, and Th cells. IFNγ induced by Th cells promotes MΦ phagocytosis. The main transcriptional mechanism in Th1 cells is TBX21 (Zhu and Paul, 2008).

TBX21 belongs to a group of genes that share the T-box. In mice, TBX21 regulates the secretion of IFNγ. This finding suggests that TBX21 plays a crucial role in the maturation of naïve Th cells by regulating the secretion of IFNγ. In naïve Th cells, TBX21 can be induced through two independent signaling pathways, IFNγ/STAT1, and IL-12/STAT4, but both signaling pathways must be induced to reach a stable Th1 phenotype. Additionally, Th1 cells are stabilized by inhibiting maturation to Th2 and Th17 cell phenotypes. TBX21 promotes Th1 cell-mediated immune responses and suppresses immune responses of Th2 and Th17 cell phenotypes. TBX21 also affects cytotoxic T and B cells. In cytotoxic T cells, TBX21 stimulates the expression of IFNγ and granzyme B and cooperates with the transcription factor EOMES to induce their maturation (Lazarevic et al., 2013). In B cells, TBX21 elicits a Th1 cell immune response, induces the secretion of antibodies IgG1 and IgG3, and induces memory B cells that elevate the expression of the immune response upon viral infection. These cells induce IFNγ expression and differentiate naïve Th cells into a Th1 phenotype *in vitro* (Knox et al., 2019).

Pearce et al. (Pearce et al., 2003) demonstrated that EOMES, found in activated CD8^+^ T cells, promotes IFNγ expression similar to TBX21. This led the authors to speculate that EOMES could “complement the role of TBX21 in regulating cellular immunity by providing redundancy and possibly cooperativity to effector gene expression in T and NK cells.” Therefore, it was found that TBX21 and EOMES have antagonism different signaling pathways and could be induced through competition for transcription modifiers. TBX21 can also be induced by MYD88, which is downstream of TLR9 signaling. The mechanism by which TBX21 is stimulated is also involved in other lymphocyte populations that produce IFNγ. TBX21 is also present in innate lymphocyte cells, γδ T cells, NK cells, and NK T cells. IFNγ can be secreted by innate lymphocyte cells, γδ T cells, and CD4^+^ and CD8^+^ T cells during viral infections (Szabo et al., 2002; Lugo-Villarino et al., 2003; Kwong et al., 2017; Pritchard et al., 2019).

A recent study using a vaccination model also demonstrated that TBX21 plays a critical role in the maturation of IFNγ-producing follicular B helper T cells (Fang et al., 2018). The Winslow laboratory was the first to link TBX21-expressing B cells to pathogen-induced responses. Through a mouse model of *Ehrlichia muris* infection, they confirmed the emergence of memory B cells expressing TBX21. These memory B cells are essential for stimulating IgG secretion against secondary attack and myeloid IgM^+^ antibody-secreting cells, which can be generated from IgM^+^ TBX21^+^ precursors, thus protecting against lethal *E. muris* infections (Racine et al., 2011; Yates et al., 2013).

Isoprinosine also induces the expression of NF-κB through MYD88, which is downstream of TLR9 signaling in B cells (Peng et al., 2016). TLR9 is expressed in monocytes, B lymphocytes, NK cells, and plasmacytoid DCs. TLR9 signaling leads to B cell activation and the activation of cells that initiate a pro-inflammatory response that produces cytokines, including type I IFN, IL-6, tumor necrosis factor (TNF), and IL-12 (Martínez-Campos et al., 2017). NF-κB is a transcription factor that plays a crucial role in orchestrating innate and adaptive immune responses. It is activated through an independent signaling component when the T or B cell receptor is activated. Activated NF-κB enters the nucleus and stimulates gene expression in T cell maturation and proliferation (Lawrence, 2009). Thus, isoprinosine upregulates the immune response via NF-κB expression.

In addition, isoprinosine promotes the expression of co-stimulatory molecules, eliciting cellular and humoral immune responses that affect long-term immune responses. It induces T cell differentiation and maturation by promoting the expression of the co-stimulatory molecules CD80 and CD86, which bind with CD28 on T cells. It also promotes B cells by inducing the expression of CD19, CD21, and CD81.

Isoprinosine has also been reported to alter the course of the disease during the initial treatment of COVID-19 infection. This immunomodulatory function of isoprinosine provides critical evidence for applying isoprinosine as a FMD vaccine adjuvant (Beran et al., 2021).

In summary, isoprinosine induces the expression of transcription factors (*NF-κB*, *MYD88*, *STAT1*, *STAT4*, *TBX21*, and *EOMES*), cytokines (*IFNα*, *IFNβ*, *IFNγ*, *IL-6*, *IL-1β*, *IL-12p40*, *IL-23p19*, *IL-23R*, and *IL-17A*), and co-stimulatory molecules (*CD80*, *CD86*, *CD28*, *CD19*, *CD21*, and *CD81*), by stimulating pattern-recognition receptors (*RIG-I* and *TLR9*), thereby eliciting a potent cellular and humoral immune response that results in host defense and long-term immunity (**Figure 6**). A challenge experiment was conducted to evaluate the host defense against FMDV infection with an isoprinosine-containing FMD vaccine, including control groups (PC and NC). As a national agency of the Republic of Korea, our Animal and Plant Quarantine Agency sets goals in accordance with the policies promoted by the government. For one of our goals, “local (domestic) production of FMD vaccines”, we are developing an FMD vaccine that can replace the currently imported FMD vaccines. Therefore, FMD vaccine candidates that showed significant efficacy in target animal (pig) experiments will be selected, and host defense capabilities will be compared with imported vaccines through pig challenge experiments. The spectrum of commercial vaccine-mediated protection is narrow because commercial vaccines containing O Primorsky antigen+A Zabaikalski antigen failed to induce VN titers to protective levels against O PA2, A YC, O JC and A GP. Therefore, it is presumed that even with the same serotype (type O), cross-protection was difficult and did not protect the host from the O JC challenge. The vaccine containing isoprinosine proved capable of host protection against FMDV infection. Considering the results of the SP ELISAs and VN test performed on pigs administered the vaccine containing isoprinosine, the new FMD vaccine containing isoprinosine is expected to achieve host defense against FMDV type A infection. These results demonstrated that the immune response induced by isoprinosine induces host protection by inhibiting FMD viral replication in pigs (**Figure 7**). It can be concluded from these results that isoprinosine stimulates the key players in innate immunity (MΦs, DCs, and unconventional T cells) to achieve initial host defense and significantly enhance subsequent adaptive immunity. In conclusion, the novel FMD vaccine containing isoprinosine could serve as an axis of cellular and humoral immune responses that orchestrate not only FMD but also various other livestock diseases (*e.g.*, African swine fever and porcine reproductive and respiratory syndrome), for which there is currently no commercial vaccine. Future studies may be conducted to use this new FMD vaccine adjuvant, especially the immunomodulator, as a blueprint for developing commercial vaccines for these difficult-to-control livestock diseases.

## 5 Conclusions

Although commercial FMD vaccines have been developed to prevent viral infection, several drawbacks persist, including local side effects at the vaccination site, low antibody titers, and uncertain host defenses. We have formulated a novel FMD vaccine to address these issues by incorporating the immune-enhancing agent isoprinosine into the conventional FMD vaccine.

Through experiments involving both experimental animals (mice) and target animals (pigs), we have substantiated that the novel vaccine containing isoprinosine surpasses its counterpart lacking isoprinosine in terms of inducing antibody titers and virus-neutralizing antibody titers. We have also demonstrated that our vaccine is more effective in eliciting host defense against FMDV infection compared to commercial vaccines. Furthermore, we have uncovered the immune-enhancing mechanisms triggered by FMD vaccines containing isoprinosine in pigs. Our study offers fresh insights into FMD vaccine development strategies and underscores the potential of isoprinosine as an immune enhancer.

## Data availability statement

The original contributions presented in the study are included in the article/**Supplementary Material**. Further inquiries can be directed to the corresponding author.

## Ethics statement

The animal study was approved by Animal and Plant Quarantine Agency (APQA). The study was approved by the ethics committee of the APQA (certification no.: IACUC-2022-658). The study was conducted in accordance with the local legislation and institutional requirements.

## Author contributions

HWK: Formal analysis, Investigation, Software, Validation, Visualization, Writing – original draft, Writing – review & editing. M-KK: Investigation, Writing – original draft. SS: Investigation, Writing – original draft. SHP: Investigation, Writing – original draft. J-HP: Resources, Writing – review & editing. S-MK: Resources, Writing – review & editing. MJL: Conceptualization, Formal analysis, Funding acquisition, Investigation, Methodology, Project administration, Resources, Software, Supervision, Validation, Visualization, Writing – original draft, Writing – review & editing.
