# Supplementary material for: Isoprinosine as a foot-and-mouth disease vaccine adjuvant elicits robust host defense against viral infection through immunomodulation
Source: Front Cell Infect Microbiol. 2024 Mar 6;14:1331779. doi: 10.3389/fcimb.2024.1331779 (PMC10951065; doi:10.3389/fcimb.2024.1331779)

*Supplementary Material*

# Isoprinosine as a foot-and-mouth disease vaccine adjuvant elicits robust host defense against viral infection through immunomodulation

**Hyeong Won Kim[1], Mi-Kyeong Ko[1], Seokwon Shin[1], So Hui Park[1], Jong-Hyeon Park[1], Su-Mi Kim[1], Min Ja Lee[1]***

[1]Center for Foot-and-Mouth Disease Vaccine Research, Animal and Plant Quarantine Agency, 177 Hyeoksin 8-ro, Gimcheon-si, Gyeongsangbuk-do 39660, Republic of Korea

*** Correspondence:**
Min Ja Lee
herb12@korea.kr

**Supplementary Table legends**

**Supplementary Table 1. List of primer sequences for qRT-PCR.**

frontiers

## Supplementary Figure legends

**Supplementary Figure 1. Cytotoxicity of isoprinosine measured by cell viability assay in BHK-21, LF-BK, ZZ-R, murine PECs and porcine PBMCs.**

**(A–E)** Cell viability of BHK-21 **(A)**; LF-BK **(B)**; ZZ-R cells **(C)**; murine PECs **(D)**; and porcine PBMCs **(E)**.

Data have been represented as the mean ± SEM of triplicate measurements (*n* = 3/group).

Statistical analyses were performed using one-way ANOVA with Dunnett's *post hoc* test.

**Supplementary Figure 2. Isoprinosine alone-mediated host defense in early stage of FMDV infection on mice.**

C57BL/6 mice (6–7 weeks-old, *n* = 5/group) were administered intramuscularly an isoprinosine alone. Mice were challenged with FMDV O (100 $LD_{50}$ O/VET/2013) or FMDV A (100 $LD_{50}$ A/Malay/97) at 3 or 7 days post-injection (dpi) using an intraperitoneal injection. Survival rates and body weights were monitored for 7 days post-challenge (dpc) with the respective viruses. **(A–E)** experimental workflow **(A)**; survival rates in 3 dpi challenged group with O/VET/2013 **(B)** or A/Malay/97 **(C)**; changes in body weight 3 dpi challenged group with O/VET/2013 **(D)** or A/Malay/97 **(E)**; survival rates in 7 dpi challenged group with O/VET/2013 **(F)** or A/Malay/97 **(G)**; and changes in body weight 7 dpi challenged group with O/VET/2013 **(H)** or A/Malay/97 **(I)**. Data are presented as mean ± SEM of triplicate measurements (*n* = 5/group).

**Supplementary Figure 3. FMD vaccine containing isoprinosine-mediated antibody titers by SP O ELISA using PrioCheck™ kit in pigs.**

For the challenge experiments, FMDV type O and type A antibody-seronegative pigs (8–9 weeks old, *n* = 5–6/group) were administered FMD vaccine including FMDV type O (O PA2) and type A (A YC) antigen (15+15 μg/dose/mL, one dose for cattle and pig use) with Isoprinosine (1 mg/dose/pig), ISA 206 (oil-based emulsion, 50%, w/w), 10% $Al(OH)_3$, and 150 μg Quil-A. One milliliter vaccine was prepared as a single dose and introduced into the animals via intramuscular (I.M.) injection. The positive control (PC) group and negative control (NC) group of pigs were treated with an equal volume of commercial FMD vaccine (O Primorsky+A Zabaikalski, ARRIAH-VAC® by FGBI "ARRIAH", Vladimir, Russia) and PBS, respectively, via the same route. Blood samples were collected at 0 and 28 days post-vaccination (dpv) in pigs for serological assays. Vaccinated pigs were challenged with FMDV type O (O/SKR/JC/2014) on the heel bulb at $10^5$ $TCID_{50}$/100 μL at 28 dpv. Data are represented as the mean ± SEM of triplicate measurements ($n$ = 5–6/group). Statistical analyses were performed using two-way ANOVA followed by Tukey's *post-hoc* test. $^{****}p < 0.0001$.

**Supplementary Table 1**

| Target | Forward/Reverse | Sequence (5'- 3') | Length (mer) |
|---|---|---|---|
| RIG-I | RIG-I F | GCACCTCATACTTACAGCCCA | 21 |
| | RIG-I R | CCACAACCAGTAGGAGCACAT | 21 |
| TLR9 | TLR9 F | TCCTCTACGACTGCATCACCA | 21 |
| | TLR9 R | GTAATTGAAGGACAGGTTGAGCTT | 24 |
| STAT1 | STAT1 F | TGCACGATGGTCTCAGCTTT | 20 |
| | STAT1 R | CAGCAGTGGGACCAAGAAGT | 20 |
| STAT4 | STAT4 F | ACATGTCAAAGCCATGTCCA | 20 |
| | STAT4 R | ATGTGACAGCCCTCATTTCC | 20 |
| MyD88 | MyD88 F | CCATTCGAGATGACCCCCTG | 20 |
| | MyD88 R | TGCACAAACTGGGTATCGCT | 20 |
| TBX21 | TBX21 F | ATCATCACCAAGCAGGGACG | 20 |
| | TBX21 R | CGTCCACGAACATCCGGTAA | 20 |
| EOMES | EOMES F | CGACTCCATGTACACCGCTT | 20 |
| | EOMES R | CGGGCTTGAGGTAAGGTGTT | 20 |
| NF-κB | NF-κB F | TCGCTGCCAAAGAAGGACAT | 20 |
| | NF-κB R | AGCGTTCAGACCTTCACCGT | 20 |
| IFNα | IFNα F | CATCTGCTCTCTGGGCTGTG | 20 |
| | IFNα R | TGAGGGGATCCAAAGTCCCT | 20 |
| IFNβ | IFNβ F | TGCAACCACCACAATTCCAGA | 21 |
| | IFNβ R | GGTTTCATTCCAGCCAGTGC | 20 |
| IFNγ | IFNγ F | GCCATTCAAAGGAGCATGGAT | 21 |
| | IFNγ R | CTGATGGCTTTGCGCTGGAT | 20 |
| IL-1β | IL-1β F | AGCCAGTCTTCATTGTTCAGGT | 22 |
| | IL-1β R | TCATCTCTTTGGGGCCATCAG | 21 |
| IL-6 | IL-6 F | CTGCAGTCACAGAACGAGTG | 20 |
| | IL-6 R | CGGCATCAATCTCAGGTGCC | 20 |

**Supplementary Table 1 (continued)**

| Target | Forward/Reverse | Sequence (5'- 3') | Length (mer) |
|---|---|---|---|
| IL-12p40 | IL-12p40 F | GGAGTATAAGAAGTACAGAGTGG | 23 |
| | IL-12p40 R | GATGTCCCTGATGAAGAAGC | 20 |
| IL-23p19 | IL-23p19 F | CCATATCCAGTGCGGGGATG | 20 |
| | IL-23p19 R | AGGCCTTGGTGGATCCTTTG | 20 |
| IL-23R | IL-23R F | TCCCTCATTGCAAAGCACAA | 20 |
| | IL-23R R | GCATCTCCTCTTGCAAGCAAAT | 22 |
| IL-17A | IL-17A F | CTCGTGAAGGCGGGAATCAT | 20 |
| | IL-17A R | GGTGTGCTCCGGTTCAAGAT | 20 |
| CD80 | CD80 F | TCAGACACCCAGGTACACCA | 20 |
| | CD80 R | GACACATGGCTTCTGCTTGA | 20 |
| CD86 | CD86 F | TTTGGCAGGACCAGGATAAC | 20 |
| | CD86 R | GCCCTTGTCCTTGATTTGAA | 20 |
| CD28 | CD28 F | TCAAAGGAGTTCCGGGCATC | 20 |
| | CD28 R | CTGAAGCAGGCGGGAGTAAT | 20 |
| CD19 | CD19 F | GGACGACAGACTTCCTGAGC | 20 |
| | CD19 R | GTTCTGGCCCATCAGGATTA | 20 |
| CD21 | CD21 F | TGCCATGCCTACAAAGCTGA | 20 |
| | CD21 R | GTAGTAACCAGGGCGGCATT | 20 |
| CD81 | CD81 F | TCAACAAGGACCAGATCGCC | 20 |
| | CD81 R | GAGCGTCTCGTGGAAAGTCT | 20 |
| HPRT | HPRT F | CCCAGCGTCGTGATTAGTGA | 20 |
| | HPRT R | GCCGTTCAGTCCTGTCCATA | 20 |

**Supplementary Figure 1**

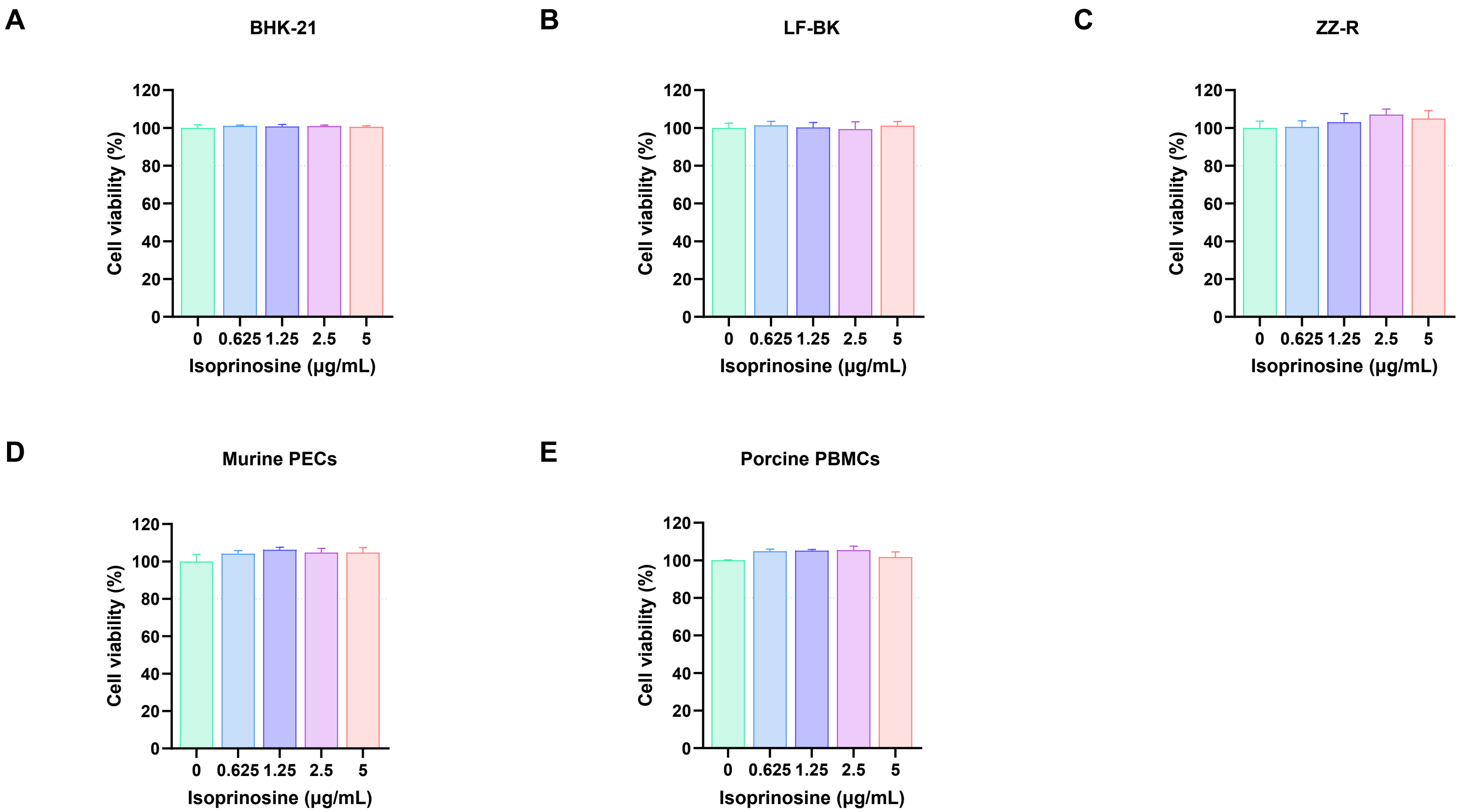

frontiers

## Supplementary Figure 2

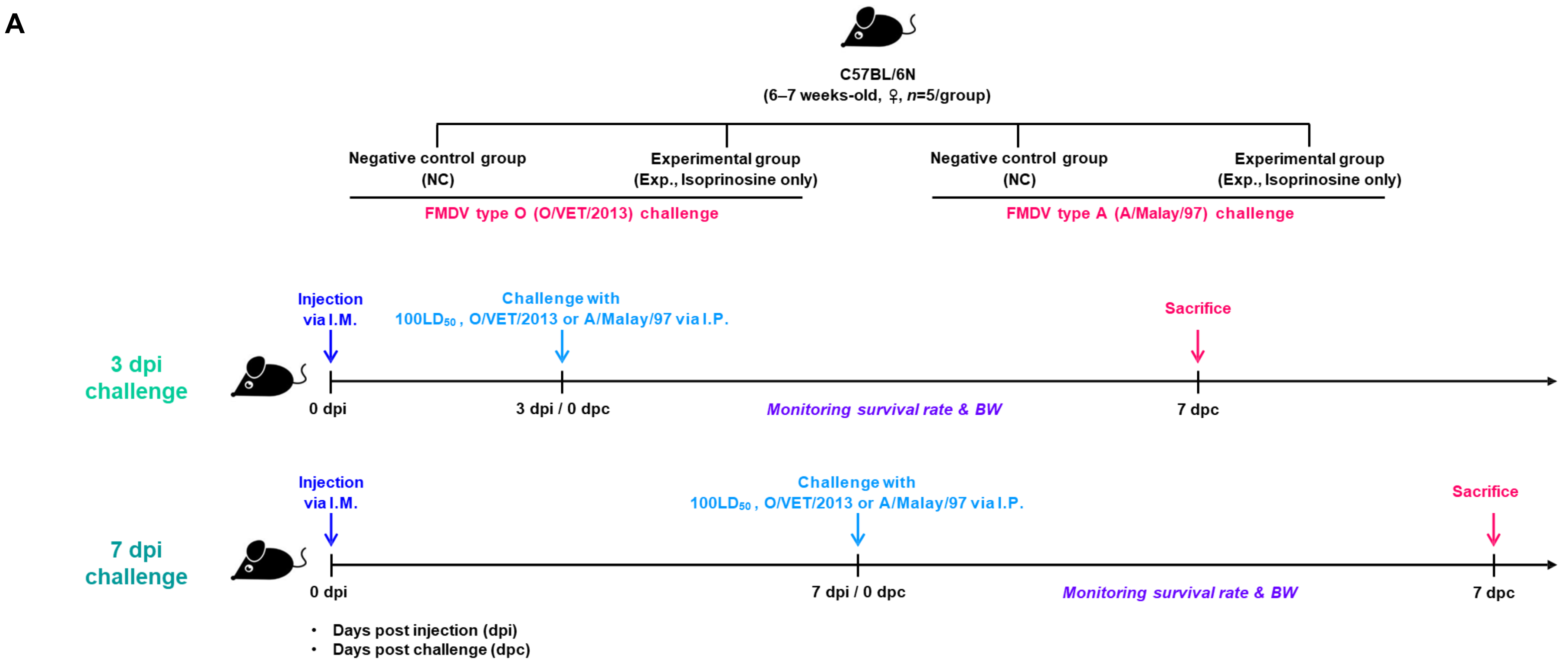

**Supplementary Figure 2 (continued)**

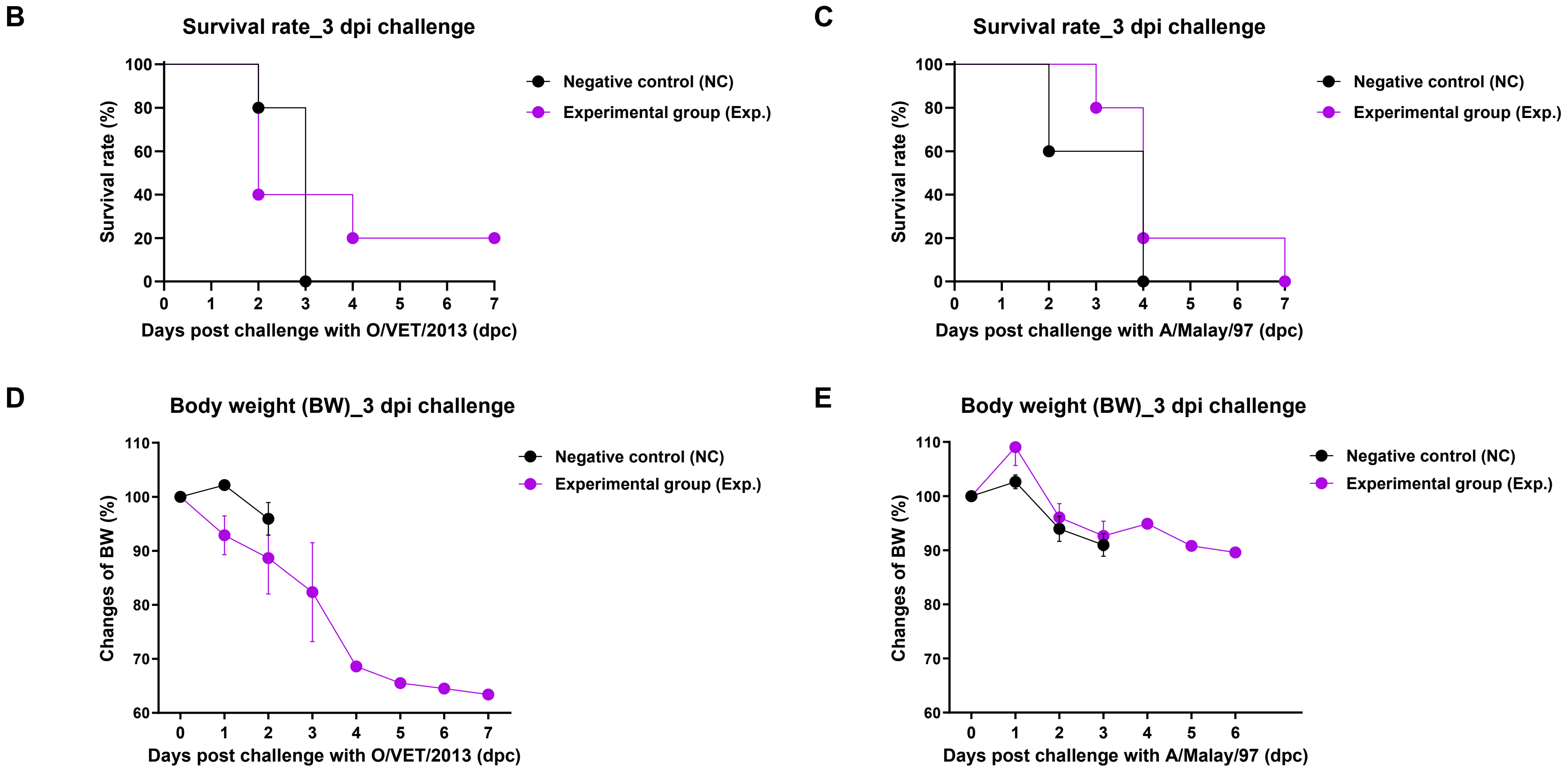

## Supplementary Figure 2 (continued)

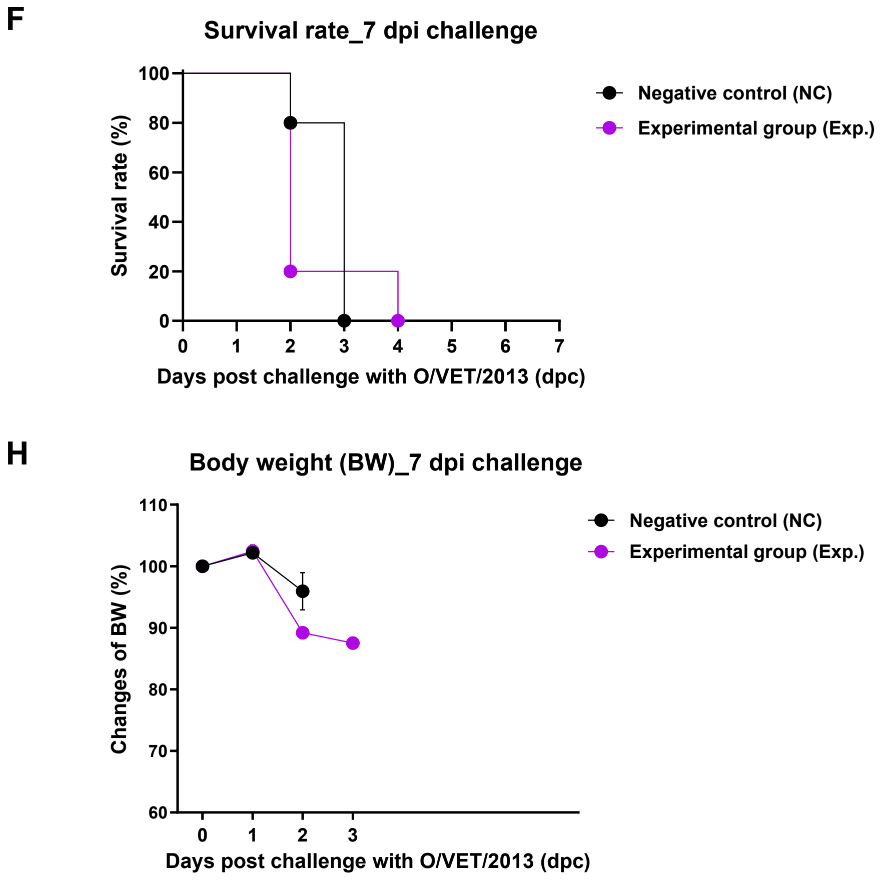

## Supplementary Figure 3

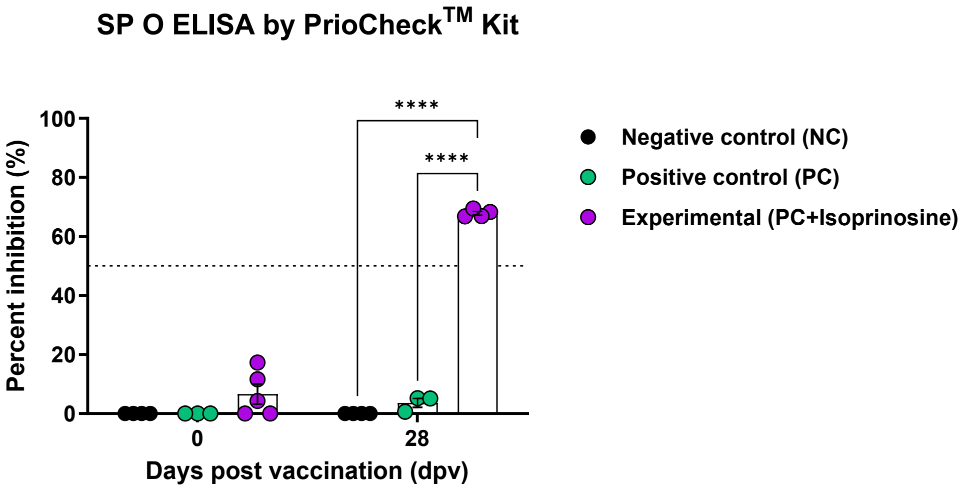

Supplement: Supplementary file 1 [file DataSheet_1.pdf]
